# Perspectives and Concerns on Late Effects Regarding Sexuality among Adolescents and Young Adults Treated for Testicular Germ Cell Tumor: The PRICELESS-Study—A Qualitative Study

**DOI:** 10.3390/cancers16040715

**Published:** 2024-02-08

**Authors:** Stefan T. Kuiper, Daniëlle Zweers, Britt B. M. Suelmann, Richard P. Meijer, Sigrid C. J. M. Vervoort

**Affiliations:** 1 Julius Center for Health Sciences and Primary Care, General Practice & Nursing Science, University Medical Center Utrecht, 3584 CX Utrecht, The Netherlands; s.t.kuiper-7@umcutrecht.nl; 2 Medical Oncology, University Medical Center Utrecht, 3584 CX Utrecht, The Netherlands; d.zweers@umcutrecht.nl (D.Z.); b.b.m.suelmann@umcutrecht.nl (B.B.M.S.); 3 Oncological Urology, University Medical Center Utrecht, 3584 CX Utrecht, The Netherlands; rmeijer6@umcutrecht.nl

**Keywords:** testicular cancer, testicular germ cell tumor, sexuality, sexual health, late effects, chemotherapy, AYA, cancer survivors, qualitative research

## Abstract

**Simple Summary:**

Sexuality remains an unspoken topic in the consulting room regarding testicular germ cell tumor (TGCT) patients in the adolescent and young adult (AYA) age. Little is known about the perspectives and concerns of these patients regarding sexuality. The PRICELESS-study explored these perspectives and concerns through individual interviews with 13 AYA patients treated for TGCT. Interviews revealed seven interacting and interconnected themes: desire to have children, rediscovering sexuality, insecurity about sexual performance, acceptance of physical change, loss of masculinity, burden on relationship, and openness in discussing sexuality. TGCT patients face multiple changes (physical, emotional, relational, and sexual), followed by a difficult period of acceptance, after which a new phase of rediscovering sexuality appears. Insights from this study can help healthcare professionals understand the underlying mechanisms and help to define topics for instrument development to support discussing sexuality.

**Abstract:**

This study aimed to explore perspectives and concerns regarding sexuality among adolescents and young adults (AYAs) possibly experiencing late effects after testicular germ cell tumor (TGCT) treatment. A qualitative study was performed in which semi-structured interviews were held with thirteen AYAs from a center of expertise for TGCT in the Netherlands. Data were analyzed using Braun and Clark’s thematic analysis method. Seven interacting and interconnected themes were found: desire to have children, rediscovering sexuality, insecurity about sexual performance, acceptance of physical change, loss of masculinity, burden on relationship, and openness in discussing sexuality. Concerns about the desire to have children seem to play a significant role. In conclusion, TGCT patients face multiple changes (physical, emotional, relational, and sexual), followed by a difficult period of acceptance, after which a new phase of rediscovering sexuality appeared. These findings can help to make healthcare professionals aware of the underlying mechanisms and concerns about sexuality. Furthermore, insights can help to develop sexuality-themed items for a broader monitoring tool to structurally assess the late effects to support discussing sexuality.

## 1. Introduction

Testicular germ cell tumor (TGCT) is the most common type of cancer among men between 18 and 40 years old, the so-called adolescents and young adults (AYA) [1,2]. Due to the excellent response to the different treatment options [3], TGCT is a relatively treatable type of cancer with a 10-year survival rate of around 95% worldwide [4,5]. Treatment consists of a radical orchidectomy and, depending on the cancer stage, supplemented with chemotherapy, radiotherapy, and/or additional surgery (e.g., removal of lymph nodes) [6]. Subsequently, a follow-up program follows for five to ten years to identify cancer recurrence and support TGCT patients in their experienced adverse effects. The treatment, especially chemotherapy and radiotherapy, can cause various side effects [7]. These side effects can be long-term effects if they occur during treatment and may persist during the long follow-up period or late effects if they occur months or years after diagnosis or after finishing the treatment [8,9].

Since TGCT affects the reproductive organs, gonadal disorders are commonly seen, often leading to late effects such as sexual dysfunction, reduced fertility, and concerns about reproduction [7,10,11,12]. In addition, a Dutch cross-sectional study showed that “changes in sexual life” and “concerns about fertility” were the fifth most common late effects and were reported by 42% of TGCT patients [13]. Sexuality and reproduction are themes that play an important role in the specific stage of life of AYAs [14]. During this phase, sexuality is developed, sexual identity is formed, and intimate relationships are established. Additionally, problems regarding sexuality have a significant impact on the quality of life in these young patients [15,16]. Therefore, sexual health is an important part of quality of life [17]. The World Health Organization (WHO) defines sexual health as “a state of physical, emotional, mental and social well-being in relation to sexuality; it is not merely the absence of disease, dysfunction or infirmity” [18]. Early detection and monitoring of late effects regarding sexuality and patients’ sexual health are essential for adequate treatment and support [19]. However, sexuality is often an unspoken topic in the consulting room [20,21,22], despite the need for AYA cancer patients to discuss this topic and receive information and support from their healthcare providers [23,24].

Although we know that sexuality issues are a common problem for TGCT patients and affect their quality of life, little is known about the concerns and underlying mechanisms from a patient’s perspective. Therefore, this study aimed to explore the perspectives and concerns on late effects regarding sexuality among patients treated for TGCT within the AYA age. 

## 2. Materials and Methods

To obtain insights into the patients’ perspectives and beliefs about sexuality and to generate a better and deeper understanding, a qualitative study was conducted [25,26,27].

### 2.1. Sample

A purposive sample of AYAs receiving follow-up care after treatment for TGCT was selected. Patients were eligible if they (1) were 18–39 years old at the time of primary diagnosis, (2) received follow-up care, (3) had completed treatment for TGCT at least six months ago, as late effects usually become apparent months after the treatment [9], and (4) were able to speak and understand the Dutch language. To provide a broad range of perspectives, maximum variation in age, relationship status, sexual orientation, type of treatment, diagnosis, follow-up time, and presence of late effects regarding sexuality was strived for [25].

Patients were recruited from an outpatient clinic of one TGCT center of expertise in the Netherlands. A nurse practitioner (DZ) selected and approached eligible patients during (e-)consultations and gave them verbal information and a factsheet with core information about the research. Patients had a three-week consideration period. After patient approval, the researcher (SK) emailed the patient an information letter. If the patient agreed to participate, the appointment for data gathering was scheduled.

Of the thirty-one selected patients, fourteen agreed to participate, ten did not respond to the invitation, and seven declined. Reasons for not participating included feeling uncomfortable discussing this topic, not wanting to revisit the topic, or considering the topic too personal to discuss. Sampling stopped when meaning saturation was reached; we defined this as the point during data analysis when no new dimensions, nuances, or insights of issues could be found within the data and the issues were fully understood [25,28]. Meaning saturation was attained after analyzing twelve patients and confirmed with the thirteenth patient.

### 2.2. Data Collection

Data were collected between March and June 2022 through one-time individual semi-structured interviews, guided by a predefined interview guide based on literature and clinical experts familiar with TGCT [29,30]. Data collection and data analysis alternated, and insights from the analysis guided further data collection, on which the interview guide was adjusted (Table 1). One researcher (SK), a 33-year-old male nurse, conducted all interviews without prior patient relationships. Patients were given the opportunity to participate face-to-face or online through video calling, with interview locations based on patient preferences. Interviews were audio-recorded, and for online sessions, video recording was employed. Seven interviews were face-to-face; four were at the patient’s residence and three in a conference room at a university. The duration of the interviews was between 21 and 56 min (mean 39 min).

After the interview, the patient administered a short questionnaire to collect demographic characteristics. The nurse practitioner (DZ) extracted disease and treatment characteristics from the patient file. Memos were made immediately after the interview to capture the atmosphere, highlights, and reflections on the interview [25].

### 2.3. Data Analysis

Data were analyzed by two researchers (SK, DZ) according to the six phases of thematic analysis of Braun and Clarke and followed an iterative process [31].

First, the recorded interviews were transcribed verbatim and de-identified. Transcripts and memos were transferred to a software program for qualitative research (NVivo, version 12) to support and systematically approach the analysis process [32]. Transcripts and memos were read and re-read thoroughly to obtain a sense of the data. Theoretical memos were made to capture the initial ideas for coding. Second, initial codes were generated independently by two researchers going systematically through the transcripts. Meaningful sentences or text sections were marked and coded. Third, the initial codes were collated and sorted into potential overarching themes based on similarity and relevance. Codes and themes were organized using a thematic mind map. Fourth, themes were reviewed by checking them with the coded text extracts and the entire dataset. Fifth, the themes were defined and refined. The essence of each theme was described, possible subthemes defined, and themes were given their final names. Sixth, a last analysis was carried out to produce a short but clear and coherent overview of the data related to the themes. Examples and quotes were selected to support and confirm each theme [31].

Throughout the analysis, codes were compared by two researchers (SK, DZ) and discussed in cases of discrepancies until a consensus was reached. A third researcher (SV) read interviews and codes and acted as a peer reviewer during the analysis. During joint meetings, the research team (SK, DZ, SV) worked towards consensus about the interpretations and themes. According to the constant comparison approach, interpretations were checked with new material and the existing data.

### 2.4. Trustworthiness

To enhance trustworthiness, the Consolidated Criteria for Reporting Qualitative Research (COREQ) guidelines were followed throughout the study [33]. Trustworthiness was encouraged in different ways [34]. Credibility was promoted by applying researcher triangulation during analysis. The use of peer debriefing throughout the research process, along with researcher reflexivity through memos, supported both credibility and confirmability. Confirmability and dependability were ensured using an audit trail, which captured the steps and decisions made throughout the research. To engage transferability and promote external validity, *thick description* was used by striving for sufficient patients, a diverse sample, and sufficient length of interview durations [34]. Lastly, the quality of the interviews and interview guide was improved by undergoing training in interviewing prior to the study and by conducting checks and discussions within the research team for the first two interviews (DZ, SV).

## 3. Results

Thirteen patients with a median age of 30 years (range 19–43 years) participated in the study. All patients were Dutch; most had a partner, and two were married. One patient identified as homosexual. Various cancer stages were observed, of which stage two was the least prevalent. All patients underwent a radical orchidectomy, with one undergoing a bilateral orchidectomy. Almost half of the patients received platinum-based chemotherapy (Table 2).

**Figure 1:**
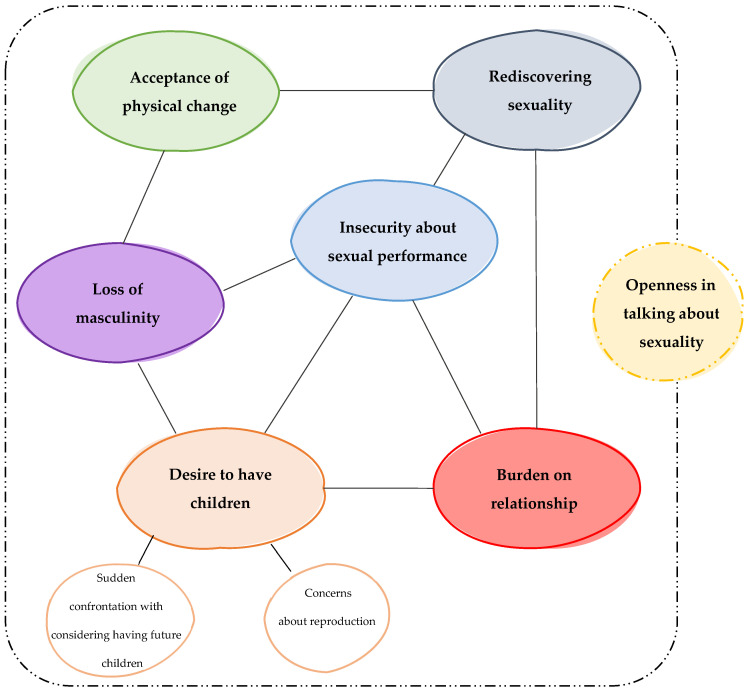
Themes, subthemes, and interconnectedness between the themes.

The thematic analysis revealed seven major themes and two subthemes: (1) desire to have children, with subthemes of sudden confrontation with considering having future children and uncertainty about reproduction, (2) rediscovering sexuality, (3) insecurity about sexual performance, (4) acceptance of physical change, (5) loss of masculinity, (6) burden on relationship, and (7) openness in discussing sexuality. The results further demonstrate the interconnectedness of the identified themes, with the theme ‘openness in discussing sexuality’ having common ground with all other themes (Figure 1).

### 3.1. Desire to Have Children

The desire to have children was the first and most common concern patients had at the time of diagnosis and was considered most important if they had the desire to have (more) children. Although there were also concerns about sexuality, patients were primarily concerned about their fertility, especially immediately after diagnosis.

*“And of course, they have also indicated that it’s possible that the chemotherapy causes your good cells, including the good sperm cells, to be damaged. And that’s why I started thinking about or worrying about fertility and not necessarily about sexuality itself.”*

(P5, 25 y/o)

Desire to have children consists of two subthemes: ‘sudden confrontation with considering having future children’ and ‘uncertainty about reproduction’.

#### 3.1.1. Sudden Confrontation with Considering Having Future Children

Directly after diagnosis and before treatment started, the patients were asked to preserve their semen to ameliorate the chance of becoming a father in the future. This discussion about semen preservation suddenly forced the men to think about having children much earlier than expected. Young patients without children did not often think about their desire to have children before diagnosis.

*“…uh, so they could freeze that [semen] and check if it was still fertile. While I didn’t worry about it at that time, but then you suddenly start thinking a lot about it (…) even if you don’t worry about it, you suddenly have to think about it. I thought that was uh… not annoying, but… weird or something that you suddenly have to think about it.”*

(P5, 25 y/o)

Patients did not find it difficult or disturbing to think about their desire to have children so early. However, thinking about their desire to have children within 12 to 48 h after the doctor told them they had cancer was, for most patients, an uncomfortable experience. Moreover, patients did not have much time to think about whether to have children, because the focus quickly shifted to cancer treatment. 

#### 3.1.2. Uncertainty about Reproduction

The diagnosis and treatment forced patients who had a desire to have children to confront the uncertainty of whether they could still have children. Uncertainty about reproduction was only mentioned by men with a current desire to have children. Semen preservation helped to create some certainty and gave them peace of mind because it was seen as a backup to have children if it turns out it is not possible the natural way.

*“…it’s nice that you have several straws of yours somewhere in the freezer, which you can use at all times.”*

(P8, 28 y/o)

Concerns about fertility remained after treatment. Patients were afraid of losing the contralateral testicle. In addition, patients were also worried about whether they would still be fertile. Some patients also experienced uncertainty about establishing new relationships, believing that fertility problems might hinder new relationships. One patient saw clarity regarding his fertility as a precondition before wanting to have a girlfriend:

*“Yeah, you’re thinking about it [sexuality], but... yeah, I’m still single so... that’s, well... [silence]... yeah, as long as I’m fertile, that’s- that’s I think the only thing that’s keeping me busy right now.”*

(P1, 19 y/o)

Despite the concerns about fertility, some patients tried to have children naturally after treatment. Others wanted clarity or certainty first and performed or asked for a fertility test. However, some patients postponed a fertility test because they feared disappointing test results. Despite receiving a positive outcome from the fertility test, some men experienced a lingering sense of uncertainty. Concerns about fertility were less prevalent in patients who already had children, as their desire to have children was either fulfilled or it was fine with them if it was not possible to have more children.

In the case of (possible) infertility, both the men and their partners accepted being dependent to some extent on alternative ways to fulfill their (future) desire to have children. It was seen as “there is no other way”, and they were lucky to have a plan B.

*“No, I’m not stressed about it or anything. We already had a son. So, I was like: “Well… if I’m not fertile anymore, then so be it. And if we want to and have the capacity to conceive a second one, um… then I’m okay with that”.”*

(P10, 32 y/o)

However, patients preferred conceiving children the ‘natural’ way. Some patients explained that having children through fertility treatments was seen as too burdensome for their partners.

*“So, once it can be done naturally, it would be a nice bonus. I really want that because you prefer not to enter such a [fertility] program.”*

(P2, 30 y/o)

### 3.2. Rediscovering Sexuality

All patients went through a process of rediscovering their sexuality as a result of the mental and physical changes after diagnosis and treatment. Yet, sexuality was still an important aspect of the relationship. It was seen as a means of investing in each other and feeling closer together. Furthermore, patients saw it also as part of being young. In addition, sexual problems should not exist when you are young. The way and extent to which patients rediscovered their sexual life was strongly dependent upon whether they experienced permanent sexual dysfunction. 

Patients were not concerned about sexuality at the time of diagnosis, as concerns were more focused on their diagnosis, treatment, and fertility. However, some patients did experience concerns during the process of rediscovering sexuality. A few worried about whether having only one testicle or a change in their scrotum’s feel and texture would influence their sexual experience and that of their partner.

*“Yes, uncomfortable [first time having sex], a little bit for her too. Especially because then, well, she also looks at it of course and then we shower and then... the other ball, in the beginning especially she found it still a bit scary.”*

(P9, 27 y/o)

Other patients became concerned only later when faced with decreased libido or adverse experiences during sex, such as numbness of the scar or groin area, phantom pain, pain or cramping, absence of ejaculation, and/or erectile dysfunction.

Some patients rediscovered sexuality by masturbating before becoming sexually active with their sex partners. However, sexual activity was often postponed. Sometimes, this was to allow the post-surgery wounds to heal. Sometimes, they did not feel mentally ready for sex because they were still processing the impact of cancer on their lives.

*“I think stress in particular and the impact of the uh... diagnosis and the treatment involved has greatly influenced delaying our... sexual... moments.”*

(P10, 32 y/o)

For most patients, the changes after treatment had little impact on how sex was experienced. They experienced having sex for the first time after diagnosis as pleasant and nice and fulfilling their desire for physical contact. Some patients in a relationship said that having sex felt the same as before. Being able to discuss issues with their partners in instances when something unpleasant happened during sex made rediscovering their sexuality feel familiar and pleasant. Other patients experienced having sex the first time as a bit uneasy or tense because they and their spouse were unsure if something unpleasant or unexpected might happen during sex (e.g., bleeding or pain). In addition, some men also mentioned that having sex made them uneasy because it had been a long time since they last had sex.

*“It’s like reliving it again, just discovering each other’s bodies or something. (…) It kind of felt like I had to guide her a little.”*

(P10, 32 y/o)

However, patients reported that time was needed to adjust to and accept physical changes because they evoked a feeling something was wrong (e.g., occasional cramping during sex) in the first place. Most patients succeeded in accepting their physical changes after a while. They accepted that it just belonged to them. Moreover, in their opinion, not much was changed sexually compared to before and after diagnosis.

This was not the case for patients with a decreased libido and/or erectile dysfunction, as they discovered this had a significant impact on their sexual well-being and significantly changed the way they experienced sex. They explained they could easily live without having sex, as sex no longer followed spontaneously or casually from arousal. These men dealt with this by pushing themselves or reminding themselves to have sex occasionally in order to meet their partner’s sexual needs.

*“Just like I say, when I do it now, I really have to think about… yes… push yourself… (…) yes, push yourself, to pick it up or think about it.”*

(P6, 39 y/o)

Some patients also searched for ways to improve libido, such as going to the gym to increase testosterone levels, or with medical interventions, such as testosterone gel. One patient who used testosterone gel explained that his sexual life had become dependent on it and made it possible to have sex again, although it still felt different.

*“…the testosterone gel actually determines your whole life. Or at least your sexual life…”*

(P6, 39 y/o)

Some men with sexual dysfunction mentioned that affection became more important because it was seen as an affirmation of “it’s alright”.

### 3.3. Insecurity about Sexual Performance

Some patients experienced insecurity about their sexual performance caused by decreased libido and erectile dysfunction. Because these patients did not perform the same sexually as they had before the diagnosis, men said they lost confidence in their bodies. Moreover, sex also became less frequent, which also affected their confidence. In addition, erectile dysfunction also increased the pressure to perform, which focused attention on the erection during intercourse. One patient decided to use erection pills while single to perform intermittently in bed and to feel less insecure. This method proved unsustainable when he entered a relationship because sex became more frequent. Performing well gave him self-confidence for the next time.

*“That-that, yes, if it brings uncertainty, let me put it this way, it’s just difficult (...) because then it gets into your head… (...) …and that doesn’t work.”*

(P2, 30 y/o)

Some men reported that trying to meet their partner’s sexual needs or produce children increased the pressure to perform. This sometimes led to sex with their partner feeling forced or compulsory and decreased the sense of relaxation during sex.

*“Look, when I do it now [having sex], it’s really forced. Because it has to, I have to perform, I have to, I have to (…) It’s not emptying my mind being busy with that, no. It is, [I] have to, have to perform. Well, if that happens, then, yes…. then sometimes it just doesn’t work, no.”*

(P2, 30 y/o)

### 3.4. Acceptance of Physical Change

Due to the orchidectomy and sometimes supplemented surgery (lymph node dissection), the patient’s body changed physically. Some men needed time to become used to their new appearance because their scrotum looked flat and felt different. Sometimes, patients felt shame and insecurity about what others might think. Insecurity about their altered physical appearance also sometimes manifested in difficulty having sex for the first time. One patient found it very difficult to accept his physical change. He did not want others to see or touch his scrotum because he did not want to be confronted with his physical change. Consequently, this resulted in a decreased libido and reluctance in his sexual relationship with his partner.

*“Uh, however, it isn’t nice at all if anyone was touching it, you know. That’s the point. I’m just thinking: “Hey, just stay away from me for a while”. Yes, you know? “Don’t touch me right now”. (…) Yes, I still have that.”*

(P13, 43 y/o)

For most patients, their physical change eventually became part of them as a person. For some patients, opting for a prosthesis was not an option, while others initially considered opting for a prosthesis but later changed their minds. Patients indicated they did not want to undergo additional surgery, were not bothered by the physical change, or felt their physical change no longer mattered. Moreover, they explained a prosthesis had no added value as it would feel different or unnatural. Patients also explained that their genital area is not a visible body part. Therefore, their physical change was less present than missing a tooth or breast.

*“At first, you think I’m going to do that for 100,000% anyway, and after that [treatment] it doesn’t interest you at all. (…) but it becomes a part of you so quickly that you think, “Yeah, I’m not going...” you know. It becomes so secondary because you could have been dead, and then to do something like that…”*

(P12, 27 y/o)

### 3.5. Loss of Masculinity

Patients reported a certain loss of masculinity due to the loss of their testicle, insecurity about reproduction, fertility problems, and sexual dysfunction. Patients reported that having testicles was seen as part of their masculinity and the distinction between men and women. It was a sign of manhood and something to be proud of. Losing one testicle affected their sense of masculinity.

*“Yeah… you might feel a little less uh... how to say... a little less complete or something; slightly less of a real guy. You’re actually proud of your balls and your penis, after all. (...) Yes, and if they take one away, you think, “I’m a little less man or so”.”*

(P11, 30 y/o)

Some patients said that missing a testicle gave them the feeling of failing as a man, as not being able to have children and grandchildren within the family. Furthermore, the patients with a decreased libido and/or erectile dysfunction said this greatly affected their masculinity, as they could not perform sexually as before the diagnosis.

*“Before, I was like an alpha male, and now uh… I’m a uh… little sheep. I just named something... a tame lamb. It-it’s such a world of difference.”*

(P2, 30 y/o)

### 3.6. Burden on the Relationship

A few patients experienced a burden on their relationship because of the desire to have children as well as their sexual dysfunction. The desire to have children created pressure on the relationship due to men’s reduced fertility or uncertainty about their fertility. The uncertainty of whether their desire for children would be fulfilled caused a psychological burden within the relationship. Some patients also reported that trying to get their wife pregnant increased the pressure on the relationship because of the need to perform.

*P: “For 90%, it’s gone [confidence in his body].”*

*I: “Yes, gosh (silence). That’s quite something.”*

*P: “Yes, if you are in a relationship and eventually want to have children.”*

(P2, 30 y/o)

Sexual dysfunction also burdens the relationship because patients feel they are failing their partners as they are unable to meet their partner’s sexual needs. In addition, patients sometimes experienced a lack of understanding from their partners, or their partners were sometimes insecure, thinking the sexual dysfunction had something to do with them. One patient explained he tried to compensate for the lack of sexual activity, for example, by cooking delicious meals for his partner. Moreover, he told his partner that he would understand if his partner sought sexual activity elsewhere.

*“Yes, she also has her needs, and it’s just part of it [relationship]. (…) Yes, then you’re afraid you’re failing the other [partner].”*

(P6, 39 y/o)

In contrast, patients also reported that their relationships became stronger after the diagnosis, as they felt more connected and able to rely on each other.

### 3.7. Openness in Discussing Sexuality 

Most patients were not ashamed to talk about sexuality in general. However, they would not bring up the subject themselves, but if someone else asked about it, they were open to talking about it. Patients mentioned it is relatively easy to talk about sexuality with their partners or their friends. One man indicated that he liked it when friends showed interest and asked questions about whether he could still get an erection. Some patients find that there is still a certain taboo on talking about sexuality, which makes it challenging to broach the subject, also in conversations with their healthcare professional.

*“And certainly, yes, it still remains a sensitive topic or so. Or a, uh, bit of a taboo. That you talk about that with people...(…)Yeah, because it’s still [something] with your sexuality and stuff. It’s not necessarily a taboo for me when I talk about it with friends, but it’s not something that I just, that I will immediately bring up myself.”*

(P5, 25 y/o)

Patients who were still single were slightly more reserved toward their bed partners when it came to discussing sexuality related to TGCT. Most did not share that they only had one testicle before having sex. It was indicated that it would become overly prevalent or too much trouble. Some shared their diagnosis only after sex, or when the relationship became serious.

*“Actually, I only told sometimes when I thought this is going to be a serious one [girlfriend], so then I want to tell it. So, I didn’t even really tell anyone every time I started dating if it might last maybe a week or two weeks. I thought, yeah, you don’t deserve to know it yet.”*

(P7, 30 y/o)

Healthcare professionals did not typically mention sexuality usually in conversations with their patients; sometimes sexuality was brought up by patients themselves. Patients found it positive if their healthcare provider brought up sexuality. They think it helps them broach this topic, reassures them, and might help them recognize problems earlier. Not all patients had this need and preferred to discuss it elsewhere, such as with their partner or a psychologist. Moreover, one patient mentioned involving their partner in the discussion as beneficial because his partner was also involved in the disease process, and sexuality also impacted them.

*“P: I think it’s a little easier to say yes. Even though my wife and I talk about it very openly, and she knows it all, to someone else, it’s a kind of a bridge too far sometimes.”*

*“I: Yes, it is indeed a big step to bring it up yourself.”*

*“P: yes, and if someone else says, “Oh, this and this happens very often”, “does that bother you, if not you do…”, I don’t know. I think that will help.”*

(P11, 30 y/o)

## 4. Discussion

The results of the PRICELESS-study provided valuable insights into the perspectives and concerns on late effects regarding the sexuality of AYAs treated for TGCT. Seven interacting and interconnected themes and two subthemes derived from the data: desire to have children, with subthemes of sudden confrontation with considering having future children and uncertainty about reproduction, rediscovering sexuality, insecurity about sexual performance, acceptance of physical change, loss of masculinity, burden on relationship, and openness in discussing sexuality. 

Concerns about the desire to have children seem to play a major role, especially in patients with an active desire for children. The results show that sexuality and their related concerns cover more than just having sex. It shows a process in which patients deal with several physical, emotional, relational, and sexual changes, sometimes resulting in loss of confidence and insecurity in sexual performance.

Our findings that patients’ interest in sex returned after the disappearance of treatment side effects and fear of cancer recurrence is consistent with the qualitative study in patients after rectal cancer treatment by Ball et al. [35]. Although the older age and etiology of sexual dysfunction after rectal cancer may differ from sexual dysfunction after TGCT, the time patients are ready to have sex again is comparable. Our result insecurity about sexual performance is also seen in men with prostate cancer [36]. However, a noteworthy difference with AYAs is that men with prostate cancer are older (60–80 years). They reported a sense of calmness and acceptance as their sexual desires and libido, as well as that of their partner, changed with age [36]. These feelings were not yet present in the life stage of young men with TGCT. Moreover, patients in our study felt they were failing since they were unable to fulfill the sexual needs of their partner, which created a burden on the relationship and increased insecurity. 

Although sexuality was the focus of our study, patients mentioned many fertility-related thoughts and concerns. A qualitative study of sexual dysfunction among young adult survivors of childhood cancer confirmed this finding, with concerns about fertility also mentioned in the interviews when asked about sexual dysfunction [37]. The study of Ussher et al. [38] confirms that sexual functioning and infertility are often mixed up. 

Uncertainty about fertility is commonly seen in AYA men with cancer [37,39]. Among TGCT patients in our study, uncertainty about fertility is paramount because concerns around fertility are quickly triggered by the discussion of semen preservation immediately after diagnosis and before initial treatment (orchidectomy). Moreover, patients usually had to decide within 48–72 h of diagnosis because of the planned surgery. This short time forced them to think about their desire to have children first, which is usually not the case with most other cancer diagnoses, where the waiting time for surgery is often longer. 

Our study shows that concerns about infertility affect more than a patient’s desire to have children. It also influences feelings of insecurity, establishing future relationships, and puts pressure on existing relationships, which aligns with the study of Ussher et al. [38] about fertility-related distress after cancer in young single and partnered men. They also found that fertility issues made men feel “half a man”. This is comparable to our finding that men felt a certain loss of masculinity because of insecurity about their fertility. An observational study in testicular cancer patients about sexuality and reproductive concerns also supports our finding and found a strong association between reproductive concerns and a negative body image [40]. Body image encompasses several aspects of experiences related to one’s own body, including whether a person feels masculine [41]. 

Our finding of loss of masculinity because of testicle loss is commonly seen in TGCT patients [42,43]. It is explained as a sort of symbolic loss or feeling incomplete, which is in line with the study of Dax et al. [43]. This sometimes leads men to consider a prosthesis. Therefore, difficulty in accepting the physical change might play a role in wanting a prosthesis, as the literature shows that men who opt for a prosthesis are often concerned about self-image [44]. Reasons for not wanting a prosthesis, such as not wanting additional surgery or not seeing the added value of it, are also seen in other studies on testicle prostheses [44,45,46].

The fact that most patients indicated that sexuality is not commonly discussed with their healthcare professional is also seen in other studies with AYAs, where communication was reported as insufficient or did not fulfill patients’ needs [23,47]. However, not all patients needed to discuss sexuality, especially patients without severe sexual dysfunction. In the study of Jonker-Pool et al. [48], it appeared that more than half of the patients with testicular cancer reported a lack of information and support concerning sexuality during treatment; 67% of them still had a need for information at follow-up. In particular, patients with testicular cancer who suffered sexual dysfunction reported a high need for information and support, consistent with the results of the study by Jonker-Pool et al. [48]. Patients in our study and the literature indicated that involving the partner in education and support is essential because sexual dysfunction in men with a partner also affects the partners [49].

Follow-up care plays a vital role in the early identification and monitoring of late effects such as sexuality problems to provide adequate education and support [50]. However, sexuality is often an unspoken topic in the consulting room [20,21,22]. Early identification is crucial to adequately support patients and their partners. A Dutch study among healthcare providers that specialize in AYA care shows that the use of self-reported measurement tools reduces barriers and discomfort in talking about sexual health, and therefore problems should be identified earlier [51]. Insight into the problems by identifying them early may lead to an appropriate range of support and interventions according to existing guidelines on sexual problems, such as medication or referral to a sexologist or psychologist. 

To monitor the symptoms related to adverse effects, patient-reported outcome measures (PROMs) are often used, as these self-reported tools are the most reliable in measuring the presence and intensity of symptoms [52]. The Patient-Reported Outcomes Measurement Information (PROMIS) v2.0 Brief Profile Sexual Function and Satisfaction Questionnaire (Brief Profile SexFS) is an example of a general tool that can be used for a wide range of patients and measures sexual function and satisfaction [53]. The study by Sopfe et al. shows that this instrument is suitable for AYAs in addition to adults [54]. AYAs additionally report that it helps in removing barriers [54]. At present, no PROM specific to TGCT patients is available to assess late effects in general or sexuality. The next step, based on the findings of our study, is to develop a TGCT module of the general and widely used Utrecht Symptom Diary (USD). This valid Dutch PROM is applied to routinely assess and monitor symptoms in cancer patients [52,55]. It is a self-administered questionnaire that measures the burden on twelve predefined and self-added symptoms, asks to prioritize one symptom, and rates the patient’s overall well-being [52]. However, for TGCT patients, the USD misses specific symptoms regarding sexuality. Therefore, a tailored version of the USD will be developed to enhance the early identification and monitoring of TGCT-specific symptoms regarding sexuality.

Our study adds a deeper understanding of the perspectives and concerns about sexuality and how they are related among TGCT patients. This brings the patient’s perspective to the current knowledge about sexuality among TGCT patients. The richness and thickness of the data, together with the accomplished maximum variation in patient characteristics, gives us confidence that the results are applicable to a broad range of patients. Patients’ openness in discussing this sensitive topic also yielded rich data, which might be supported by the interviewer being of the same gender and age category as the patients. Finally, using research triangulation helped enhance the findings’ credibility and validity.

However, the results may be biased by selection bias, as patients who found the subject too personal or felt uncomfortable talking about sex did not want to participate; this resulted in a low response rate. In addition, single and non-heterosexual patients were underrepresented, which reduces the transferability of the results to these patients. Recall bias may also have influenced the findings since patients at the end of their follow-up period refer to their concerns about sexuality at the beginning of their follow-up period. Patients at the end of follow-up care usually have achieved acceptance and have already rediscovered their sexuality, which may have influenced how they remember their difficulties at the beginning of their treatment trajectory. 

Our study recommends that healthcare providers be aware of the underlying mechanism involved in concerns about sexuality. Moreover, it is essential to know what patients mean by concerns about sexuality. In addition, a strong relationship with concerns about fertility must be considered regardless of the age of the AYA patient. This helps to discuss concerns about sexuality and provide tailored education and support.

Furthermore, our findings and current literature show that fertility concerns are strongly related to establishing relationships, loss of masculinity, insecurity, and a negative self-image commonly at the start of their cancer journey. Healthcare providers must be aware of these interactions between these issues to perform adequate care. Involving the patient’s partner and providing age-specific care seems helpful in this regard.

The findings of our study are also helpful in defining topics for developing tools to support the conversation about sexuality. There is a great need for patient and partner education and support, especially for patients suffering from severe sexual dysfunction. The healthcare provider should make efforts to understand the underlying mechanisms of sexual dysfunction because the etiology should guide tailored support. 

Future research using a questionnaire based on the themes found could help to confirm and support the themes and conclusions identified, thereby increasing the generalizability of the results. It may also enrich our understanding of the complex implications of late effects on sexuality after TGCT treatment and might help in engaging patients who are less comfortable talking about sexuality. Furthermore, examining the views of patients’ partners can provide additional perspectives and insights about the influence and impact on their sexuality. In addition, a longitudinal approach can help gain insight into how sexual problems evolve. Finally, follow-up research on interventions and support strategies (e.g., education, peer groups) focused on the target group and related to the themes found can be helpful and supportive.

## 5. Conclusions

In conclusion, patients with TGCT have various experiences and concerns regarding sexuality, which are reflected in multiple interrelated themes. Patients experience physical changes as well as changes sexually, physically, emotionally, and relationally. After a sometimes difficult period of acceptance of physical changes, a new phase of rediscovering sexuality often emerges. Healthcare providers should be aware of these changes, especially at the start of the follow-up period, so they can identify problems regarding sexuality and fertility early. Early identification is crucial to responding to patients’ needs and providing adequate information and support. 

## Figures and Tables

**Table 1:** Final interview guide.

Main Topic	Questions, Probes, and Subtopics
Diagnosis of TGCT	What affect had the diagnosis of testicular cancer on your life?
Late effects regarding sexuality	What changes have you noticed related to sexuality after your treatment? - Presence of late effects - Concerns - Coping - First time having sex after treatment - Masturbation
Value of sexuality in daily life	What is for you the value of sexuality? - Influence of testicular cancer on value of sexuality - Changes
Relationships	What kind of change have you noticed in relationships in general? - Fertility - Changes within your current relationship - Talking with your partner - Coping
Discuss sexuality during consultation	Which topics related to sexuality do you think are important to talk about during the consultation? - Was it discussed? - Why are these important?

**Table 2:** Patient characteristics.

Characteristic	n (=13)
Age ^1^ (years) (mean, min–max)	30.3 (19–43)
Native country (n, %)	
The Netherlands	13 (100)
Marital status ^1^ (n, %)	
Single	3 (23.1)
Relationship/with a partner	8 (61.5)
Married	2 (15.4)
Sexual orientation (n, %)	
Heterosexual	12 (92.3)
Homosexual	1 (7.7)
Stage of cancer (n, %)	
Stage 1	6 (46.2)
Stage 2	2 (15.4)
Stage 3	5 (38.5)
Treatment type ^2^ (n, %)	
Single radical orchidectomy	12 (92.3)
Double radical orchidectomy	1 (7.7)
Chemotherapy	6 (46.2)
Radiotherapy	2 (15.4)
Additional surgery	1 (7.7)
Follow-up time ^1^ (months) (mean, min-max)	32.4 (9–58)

^1^ At the time of the interview. ^2^ Patients may receive more than one treatment.

## Data Availability

The data presented in this study are available upon reasonable request from the corresponding author. The data are not publicly available as TGCT is a rare disease, and the study sample comes from one site.
